# Supplementary figures and images for: Identification of candidate reference genes for qRT-PCR normalization studies of salinity stress and injury in Onchidium reevesii
Source: PeerJ. 2019 Apr 26;7:e6834. doi: 10.7717/peerj.6834 (PMC6487802; doi:10.7717/peerj.6834)

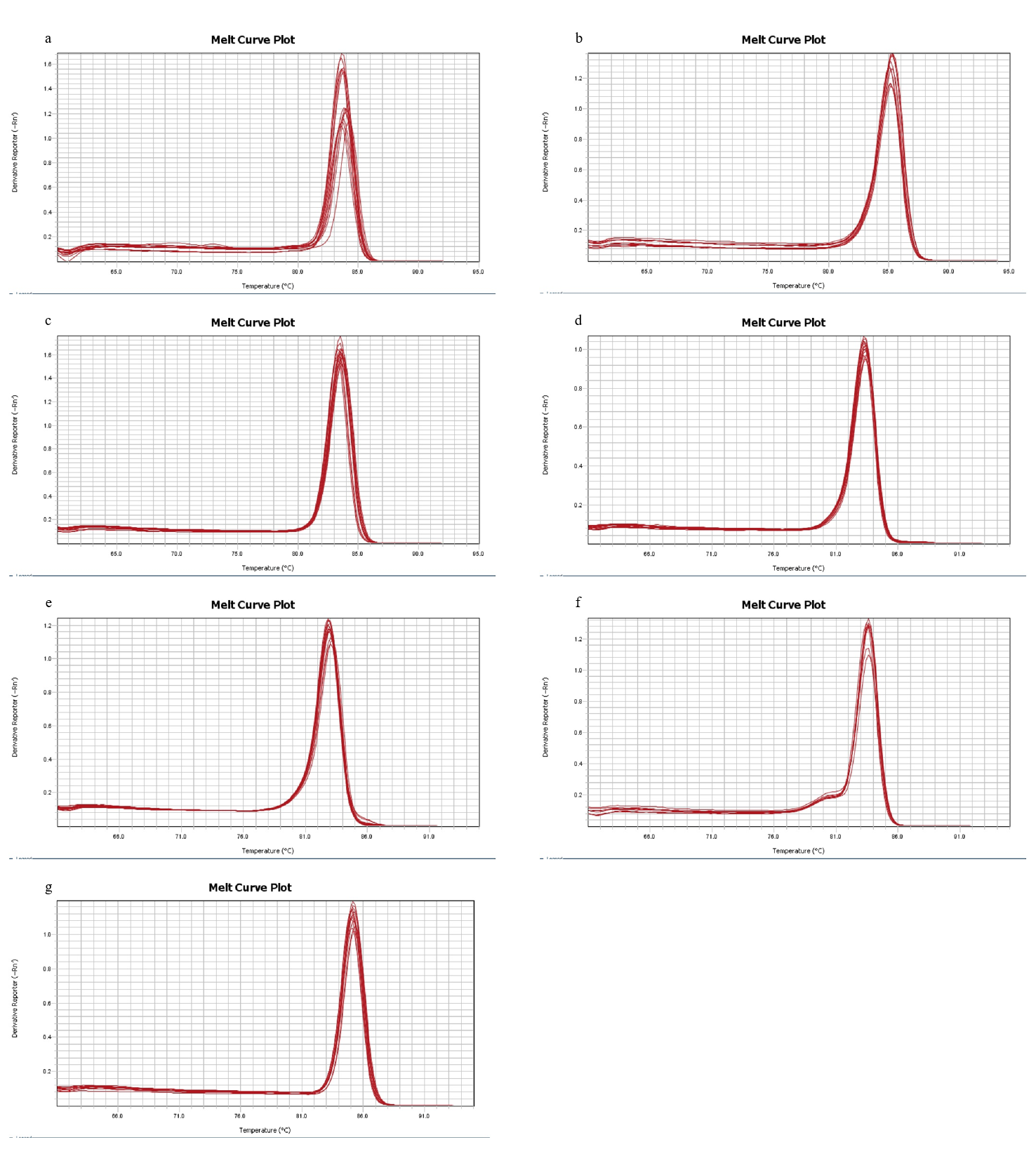

Supplement: Figure S1 — (a) to (g) represent seven candidate reference genes, CYC, RPL28S, ACTB, TUBB, EF1a, Ubiq, 18S RNA, respectively. [file peerj-07-6834-s001.png]

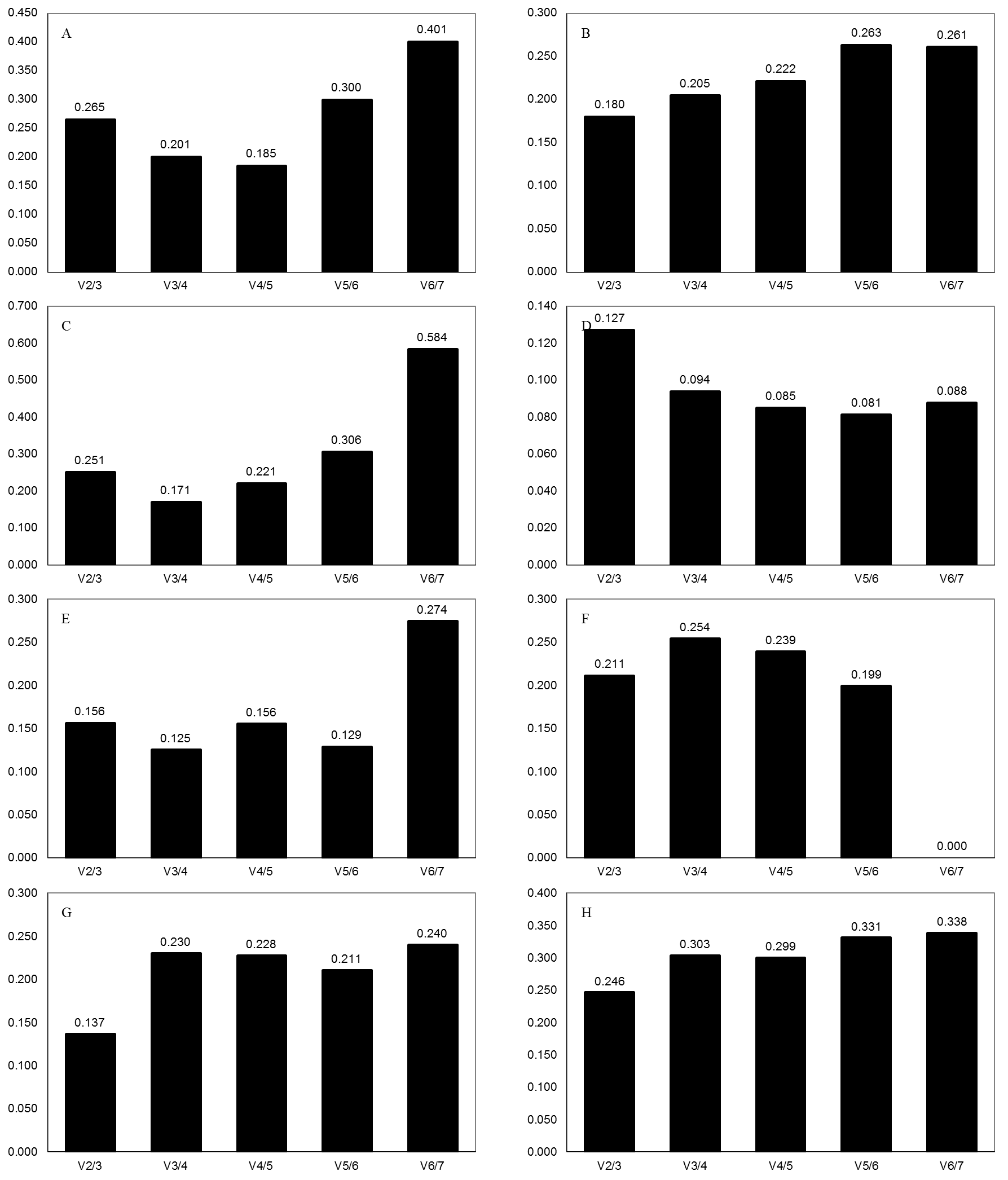

Supplement: Figure S2 — Vn/n+1 values were used to determine the optimal number of reference genes. A cutoff of 0.15 (Vn value) is usually applied. A different tissues, B dorsal muscle tissue under 0 ppt salinity; C dorsal muscle tissue under 5 ppt salinity; D dorsal muscle tissue under 15 ppt salinity; E dorsal muscle tissue under 25 ppt salinity; F dorsal muscle tissue under 35 ppt salinity; G dorsal muscle tissue after injury; H dorsal muscle tissue from animals of different weights. [file peerj-07-6834-s002.png]
